# Rare disease patients in India are rarely involved in international orphan drug trials

**DOI:** 10.1371/journal.pgph.0000890

**Published:** 2022-08-15

**Authors:** Monoswi Chakraborty, Mohua Chakraborty Choudhury, Indraneel Chakraborty, Gayatri Saberwal

**Affiliations:** Institute of Bioinformatics and Applied Biotechnology Biotech Park, Bengaluru, India; Cancer Institute Women’s India Association, INDIA

## Abstract

We wished to determine whether rare diseases patients from India had been enrolled in international trials to develop novel orphan drugs. There are two reasons to be interested in this. (a) Different ethnic or racial groups may respond differently to a particular drug. India has huge ethnic diversity, and to exclude such participants is to severely limit the diversity of any trial; (b) Even if a suitable drug for a rare disease is available in India, it may be astronomically priced, in a country where most healthcare expenditure is out-of-pocket. We identified 63 orphan drugs, approved by the US Food and Drug Administration (FDA) after 2008, for which there were 202 trials in the US government’s clinical trial registry, ClinicalTrials.gov. Only nine of these trials had run in India. These trials pertained to six drugs. The drugs were for the conditions B-cell Lymphoma, Chronic Myeloid Leukemia, Gaucher disease Type 1, Malaria, Myeloma and Pulmonary Arterial Hypertension. Further research is required as to why patients from India are not part of foreign drug development programmes for rare diseases. We then asked how many of the remaining 193 trials had recruited people of Indian origin, residing in other countries, and found that not more than 1% of these trials had done so. Also, only 11 of the 193 trials had recruited from other lower income countries. Participation from low-income countries in trials for orphan drugs is poor.

## Introduction

It is known that different ethnic or racial groups may respond differently to a particular drug. Illustratively, Caucasians require higher doses of warfarin than African-Americans [1]. And about one in five recently approved drugs in the US have exhibited different outcomes based on ethnicity [1], which may have been related to efficacy or the incidence of side-effects [2].

Therefore, it is important to ensure that the participants in trials are adequately diverse, to ensure that the outcomes of studies are generalizable, that is that they are valid in different populations. Despite several initiatives such as the Revitalization Act of 1993, which has required that trials funded by the National Institutes of Health (NIH), in the United States (US), include women and minorities [3], the diversity in clinical studies has not seen substantial improvement [4]. The recent experience of greater morbidity and mortality among minorities in the US during the COVID-19 pandemic has re-emphasized the need to increase the diversity of trial participants. Evidence of this renewed emphasis is reflected in the recent rejection of a new drug application by the US’s Food and Drug Administration (FDA), partly due to the lack of diversity in the trial population [5].

Coming to rare diseases (RDs), each RD affects very few people, although cumulatively such diseases affect large numbers of individuals. For instance, it is believed that the total number of RD patients is 29 million in Europe, 30 million in the US, and 70–96 million in India [6, 7]. Given the small number of individuals affected by a given condition, it is a challenge to conduct clinical trials [8], but the various incentives for orphan drug (OD) development have nevertheless led to many drugs and other interventions being developed.

As mentioned, pharma companies have often found it difficult to enroll participants in RD trials, due to the small size of the patient population, which is often geographically dispersed. Moreover, these diseases are often severely debilitating, which further limits the number of participants who can travel to study sites to participate. However, given that 80% of RDs have been found to have a genetic basis, it is important that RD trials are inclusive of different ethnic groups. So, the wider the companies extend their reach for patient recruitment, including internationally, the more participants they can hope to enroll in their studies.

There is also another reason to be interested in the participation of patients from India in OD trials. In general, most RDs do not have any treatment, and for those that exist, the cost may be exorbitant. Even if the treatment is available in India, it is likely to be unaffordable for most, as the patients largely have to bear these costs as out-of-pocket expenditure. As such, participation in trials is sometimes seen as the only hope to receive some treatment for diseases that may cause rapid deterioration or that may be life-threatening. Therefore it is not surprising that in previous work we found that RD patients in India are desperate to participate in global studies, and want the government to enable easy access to experimental therapies through trials [9].

To the best of our knowledge, there has been no study to analyze the participation of RD patients in India, in global trials. Here, we set out to study the number of Indian patients enrolled in a set of global OD trials. This then led to our investigation of how many people of Indian descent were enrolled in such trials, and also of how many trials were run in other low-income countries.

## Methods

**Fig 1:**
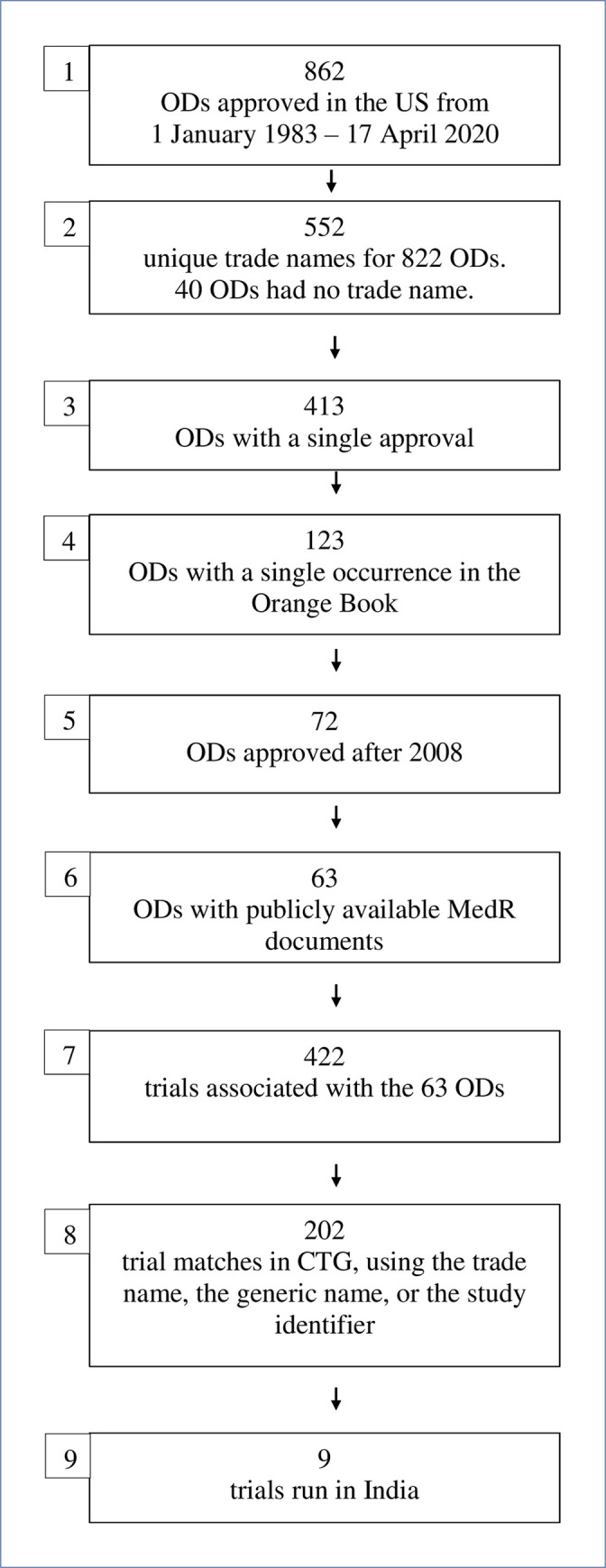
The steps involved in identifying the ODs and trials of interest. Boxes 1–6 correspond to the steps taken in the Methods section, in the ‘Data set’ sub-section. Starting with the 862 ODs, the 63 ODs that met a set of criteria were identified. Boxes 7–9 correspond to the findings in the first few paragraphs of the Results section. Starting with the 422 trials that were determined to be linked to the 63 ODs identified in Box 6, the nine trials that had run in India were identified.

This study was based on data available from the US Food and Drug Administration (USFDA) and the major public clinical trial registry in the US, ClinicalTrials.gov (CTG), as detailed below. The steps taken to identify the drugs and trials of interest are summarized in Fig 1. Each step of the methodology was performed by at least two authors, independently.

### The dataset

We accessed the FDAOD, ie the FDA’s Orphan Drug Designations and Approvals database [10] on 18 April 2020. We obtained the list of 862 ODs that had been approved from 1 January 1983 to 17 April 2020. 40 of these drugs lacked a trade name, and the remaining 822 ODs had 552 trade names overall. Of the 552, 139 had received multiple, and 413 had received a single orphan approval (S1 File). For each drug, we obtained the trade and generic names, the indication, and the marketing approval date from the FDAOD (S2 File).

Next, we wished to identify ODs that had been approved (i) for one orphan indication, and (ii) not for a non-orphan indication. To do this, we compared the list of 413 ODs with the entries in the FDA’s Orange Book, which listed every drug approved by the FDA. We accessed the Orange Book on 18 April 2020 [11]. The download from this site was in the form of three files, that is exclusivity.txt, patent.txt, and products.txt. We compared the list of 413 ODs with the list of drugs in products.txt. 123 of them had a single occurrence in the Orange Book (S1 File). We took forward this set of 123 drugs. Notably, since biological products were not included in the Orange Book [12], no biologic was included in our dataset.

We wanted to study more recently approved ODs, and therefore focused on those approved from 1 January 2009–17 April 2020 (S1 File). This reduced the number of drugs from 123 to 72. For each of these drugs, we wished to examine the FDA documents concerned with its approval. Accordingly, we looked for the Medical Review, Clinical Review, or Multidiscipline Review–all referred to as MedR henceforth–at the Drugs@FDA site [13]. A MedR was publicly available for only 63 of the 72 drugs (with further details in S1 File). Of the 63 ODs, 27 (43%) were approved from 2009–2014, and 36 (57%) from 2015–2020 (S1 File).

To summarize, we considered 63 ODs, each approved after 2008, and each with a publicly accessible MedR. Each of these drugs was approved for a single indication and was mentioned only once in the FDAOD and in the Orange Book.

### Data from the FDA MedRs

We went on to search the relevant MedR for the trials associated with a given drug. We primarily examined the table entitled ‘Table of Studies/Clinical Trials’, or ‘Table of Clinical Studies’, in the section ‘Sources of Clinical Data and Review Strategy’. Henceforth, we refer to this summary table as ‘the Table’. For most drugs, the Table provided most of the required information, which was extracted using scripts but verified manually. However, a few MedRs did not have the Table, in which case we manually searched the entire document for study information. For each trial, we obtained the study identifier from the MedR (S2 File).

### Data from CTG

Next, we searched CTG for matches of the trials listed in the above-mentioned MedRs. First, we searched CTG using the trade names of the 63 ODs. We also searched CTG with the generic names of the ODs. Finally, we used study identifiers of the clinical trials, as obtained from the relevant MedRs, to search CTG. Sometimes, the CTG record’s ‘Acronym’ or ‘Other ID’ field matched the study identifier.

## Results

In our search for trials from the 63 MedRs, we obtained a list of 422 studies (S2 File). In searching for the same trials in CTG, we obtained 202 trials, of which 151 were identified using the trade name, 47 using the generic name, and four using the study identifier (S2 File). We could not find CTG matches for 220 trials and therefore could not take these studies forward.

For each of the 202 trials, the names of the ‘Listed Location Countries’ were extracted from the CTG study records’ pages (https://clinicaltrials.gov/ct2/show/record/) using a web scraping approach in the python programming language, on 30 Jun 2021. The web scraping program used the requests, BeautifulSoup and pandas python libraries to download, target and clean the data for the required information. For each study, we obtained the NCT Number, Acronym/other IDs, and condition from CTG (S2 File).

We found that of the 202 trials underlying 63 ODs, 193 had not recruited from India, and only nine (4.5% of 202) had done so. These nine studies were concerned with the following medical conditions: Chronic myeloid leukemia (2 cases), Gaucher disease Type 1 (2), Pulmonary arterial hypertension (2), B-cell lymphoma (1), malaria (1) and myeloma (1). The two Chronic myeloid leukemia trials were for the same drug, Omacetaxine mepesuccinate, had the same sponsor, and were started within six months of each other. The two Pulmonary arterial hypertension studies were for the same drug, Macitentan, had the same sponsor, and were started within six months of each other. The two Gaucher disease trials were for the same drug, Eliglustat tartrate, had the same sponsor, and were started within seven months of each other. As such, these nine studies were related to six (9.5% of 63) drugs. Further details of each of the nine trials are reported in Table 1 and S3 File.

**Table 1:** Details of the 9 OD trials run in India.

NCT ID	Trade Name	Medical Condition Reported	Number of participants (globally)*
NCT00375219	SYNRIBO	Chronic Myeloid Leukemia (CML) With the T315I BCR-ABL Gene Mutation	103 (trial completed)
NCT00462943	SYNRIBO	Chronic Myeloid Leukemia (CML) Who Have Failed or Are Intolerant to Tyrosine Kinase Inhibitor Therapy	100 (trial completed)
NCT00660179	OPSUMIT	Symptomatic Pulmonary Arterial Hypertension	742 (trial completed)
NCT00667823	OPSUMIT	Symptomatic Pulmonary Arterial Hypertension	550 (trial completed)
NCT00891202		Gaucher disease Type 1.	40 (trial completed)
NCT01074944		Gaucher Disease	170 (trial completed)
NCT01376167	KRINTAFEL	Malaria	851 (trial completed)
NCT02227251	XPOVIO	Diffuse Large B-cell Lymphoma	244 (recruiting)
NCT03110562	XPOVIO	Multiple Myeloma	402 (active, not recruiting)

* These numbers were extracted from ClinicalTrials.gov on 28 June 2022

We then tried to identify the reason that these nine trials were run in India. For each of them, we accessed the CTG record, and searched the ’Tabular View’ of that record for any details related to a publication. The nine trials were linked to a total of 36 publications. Trial NCT01376167 related to malaria. Each of the other trials was conducted in 10 or more countries, as follows: NCT00375219 (10 countries), NCT00462943 (10), NCT00660179 (39), NCT00667823 (39), NCT00891202 (12), NCT01074944 (17), NCT02227251 (19), and NCT03110562 (21). Also, for six of the eight non-malaria trials (except NCT00462943 and NCT00667823), the results were analyzed by race or ethnicity (S3 File). No publication provided any further information that may have indicated why the trial recruited participants from India.

Next, we wished to sample the 193 trials that had not recruited participants in India, to determine whether these studies had recruited people of Indian origin, based in other countries. We used a random number generator to identify 25% (47) of the 193 trials, and examined the ‘Race’ field in the results section of their CTG records. 23 records either did not have results, or did not analyze for race. For those that did consider race, ‘Indian’ was not a category but ‘Asian’ was. The trials had different ways of categorizing race. If Asian was a category, we considered those participants. In other cases, where the categories were White/Black/Other, for instance, we considered ‘Other’. We did not consider the ‘Mixed’ race category. Based on this, we found that 214 (8%) of a total of 2664 trial participants may have been Asian. Since ‘Asian’ is a very large category, we then looked at how many of the trials had run in Asian nations. We found that 182 (85%) of the 214 participants were part of trials that had run in Hong Kong, Japan, Korea, Malaysia, Taiwan, Thailand or Vietnam.

Finally, we wished to identify any stated reasons for why particular trials had run in other low-income and lower-middle income countries. In order to understand the trials in such countries, we made a list of the countries from which recruitment took place for the 193 trials that did not recruit from India. We then identified which of them included one or more nations that were on the World Bank’s lists of Low-income and Lower-middle income countries (S4 File) (https://datahelpdesk.worldbank.org/knowledgebase/articles/906519-world-bank-country-and-lending-groups). Eleven trials (NCT00386763, NCT00709969, NCT00912093, NCT01390220, NCT01458119, NCT01529034, NCT02216123, NCT02342886, NCT03036813, NCT04002297, NCT00943111) included such countries (S4 File). These trials had a total of 23 publications for which information was provided in the trial records at CTG. We examined each of these publications (and supplementary information if accessible). Four trials were located in high-prevalence regions for malaria (NCT00386763, NCT00709969, NCT02216123) or sickle cell disease (NCT03036813). These four trials were covered in eight publications. Of the remaining, five trials (NCT00912093, NCT01390220, NCT01529034, NCT04002297, NCT00943111), that were linked to five publications, dis-aggregated their findings based on race (S4 File). No other publication provided any further information that may have indicated why the trial recruited participants from a lower income country.

## Discussion

In this study, we have focussed on data from CTG and the FDA. In principle, we could have looked at rare disease trial data from other registries, and in particular from the Indian registry, Clinical Trials Registry- India (CTRI). However, since regulatory documents from India’s Central Drugs Standard Control Organization (CDSCO) are publicly unavailable, even if we could identify the trials that were for rare diseases in CTRI–a non-trivial task–we could not have compared the trial data with the regulatory documents.

In terms of other registries, it is known that CTG is the largest public clinical trial registry [14, 15]. In 2021, in a comparative analysis of 18 public registries, 17 of which are recognized by the World Health Organization (WHO) as primary registries and one–CTG–as a ‘data provider’ [15], we determined that CTG accounted for 58% of the trials in these 18 registries. No other registry held even 10% of the records. For this reason we believed that analyzing data in CTG ought to suffice for this study.

Our study indicates that very few patients from India were enrolled in international OD trials. We sought to determine why some trials did recruit in India. Given the local prevalence of malaria, it is understandable that the trial related to malaria would do so. And given that all the other trials ran in 10 or more countries, with most of them analyzing their results by race or ethnicity, one can surmise that most of the sponsors sought to recruit patients from a range of ethnic or racial backgrounds. One can speculate that India was chosen as a location due to the potentially large number of patients.

Nevertheless, most trials did not recruit from India. We believe that possible reasons include (i) the poor RD ecosystem in India with low rates of identifying RD patients; (ii) poor linkages between the medical fraternity or patient organizations and foreign sponsors of studies; (iii) regulatory hindrances for experimental drugs to be trialed in India; (iv) the excessive costs of importing experimental drugs into India and (v) the very high cost of enabling patients to travel abroad to participate in a study. In case sponsors attempted to enroll patients from India, but did not succeed, possible reasons included (i) poor awareness of the potential benefit of participating in trials and (ii) the excessive burden on patients for participating in studies.

In examining whether the other trials recruited a significant number of participants of Indian descent, we determined that a significant fraction of Asians in these trials would not have been of Indian descent. Overall, only about 1% of the participants in trials that did not run in India may have been of Indian origin. We do not know why this number is so low other than the fact that minorities are generally under-represented in trials in the US [4]. This issue may merit further investigation.

As far as the trials run in low- or lower-middle income countries went, nine of the 11 trials were either run in countries where the condition of interest was highly prevalent, or had results that were analyzed based on race. The latter trials were clearly conscious of the need for racial or ethnic diversity, and are likely to have sought international sites in order to obtain this diversity. No publication provided any other indication as to why the trials were located in these lower income countries. Overall, very few trials were located in low- or lower-middle income countries. Some of the reasons postulated for the lack of sites in India, above, may hold good for such countries as well.

Returning to the trials that ran in India, aside from the fact that the development of any OD, anywhere in the world, could improve the diversity of trial participants by enrolling patients from India, there is another angle from which we could examine the issue, and that is the care required by these patients. In general, the RD ecosystem in India is underdeveloped. With its large population of almost 1.4 billion people, one expects that the country is host to most rare diseases or conditions. And yet patients representing only about 450 of the estimated 6000–8000 RDs have been identified in the country [7]. Since it is estimated that there are between 70 and 96 million RD patients in the country, there is much work to be done to spread awareness about such ailments, identify patients, and provide the best available care. Since many RDs may have greater prevalence in groups with high rates of endogamy or consanguinity [6, 16], such populations need special attention, and also suitable genetic counseling.

For patients whose condition has been correctly diagnosed, there may be an enormous challenge to obtain a high-priced therapeutic that is primarily available in Western nations. Although it is not uncommon to have to pay between Rs. 1 million or more for a lifetime’s treatment [7], the cost can be substantially higher. Illustratively, the annual cost–Rs. 25 million–for suitable enzyme replacement therapy for a seven-year old child with Morquio syndrome worked out to 77 times the child’s father’s annual income [17]. Although a few patients have received similarly high-priced drugs free, on compassionate grounds, through a global lottery, most have not. In early 2021, in an unprecedented feat, a crowdfunding effort raised Rs. 160 million for a child who needed a single dose of the drug Zolgensma for spinal muscular atrophy type 1 [18]. In this case, the government also waived the Rs. 50 million import duty.

The prevalence of medical insurance in the country is low, and in recent years it has been estimated that less than one in four Indians have some coverage [19, 20]. This makes it even harder for patients to access these expensive cures. The only long-term solution is to undertake relevant drug discovery and manufacture in India. This should enable (i) the prioritization of drug development for the more common among the RDs in India; (ii) a reduction in the costs of trials if patients do not have to be taken abroad to participate in them [21]; (iii) the drugs to be more affordable; and (iv) the drugs to be tailored for Indian patients, if required [22, 23]. The New Drugs and Clinical Trials Rules 2019 have outlined some regulatory benefits for ODs, such as the possibility of (a) a waiver of local clinical trials if the drug is approved in certain countries, or (b) a waiver of the fee for conducting clinical studies in India [24]. These regulatory provisions may help to speed up the availability of ODs in the country.

Finally, this study has some limitations. (a) We studied drugs approved after 2008. Since September 2007, the The United States Food and Drug Administration (FDA) Amendments Act of 2007 (U.S. Public Law 110–85) has been in effect [25]. This law required more trials to be registered with CTG. By studying drugs approved after 2008, we left a large margin for sponsors to comply with this law. We could have obtained a slightly larger sample size if we had not allowed this margin. (b) We studied a narrowly defined set of ODs. It is conceivable that the results are not generalizable to a larger set of ODs, or to non-ODs. (c) The fact that the race and ethnicity of the participants from a given site are usually not available in adequate detail means that it is possible that we have not correctly captured the number of Indian-origin participants. Also, since we only sampled 25% of the eligible trials, it is possible that we missed a significant number of Indian-origin trial participants. However we identified an extremely small fraction of Indian-origin participants, and therefore do not believe that this fraction would be significantly more even if all 193 trials were considered. (d) It is known that there are errors in trial registries [26–30]. The data in a registry is rarely audited by independent organizations, and therefore our assumption that the data regarding the location of sites or race of the participants is correct cannot easily be corroborated.

## Conclusion

In this work, we have determined that very few RD patients from India have been enrolled in international trials to develop novel ODs. For the studies underpinning the approval of 63 ODs approved in the US, trials were performed in India for only six drugs. Also, the number of Indian origin participants in the trials for the other drugs was negligible. Further, very few trials ran in other low-income countries. This is a sub-optimal situation from two points of view, ie (i) it is important to increase the diversity of participants in studies for ODs and (ii) given the very high prices of ODs, and the lack of health insurance for so many patients, RD patients in India, and probably in many other low-income countries, have limited treatment options. Participation in international studies would be one limited way to access promising drugs. Further research is required as to why more patients from India and other low-income countries are not part of such trials.

## Supporting information

S1 File The steps involved in identifying the 63 ODs for this study. a. The list of 862 ODs approved in the US between 1 January 1983 and 17 April 2020, inclusive, obtained from FDAOD. b. The list of the 862 ODs’ 552 unique trade names, and their break up into (i) 413 with single approvals, (ii) 139 with multiple approvals, and (iii) 40 cases with trade names unavailable. c. Of the 413 single approval trade names, 123 had a single mention in the Orange Book, and 290 either did not have any mention in the Orange Book or had multiple mentions in it. d. Of the 123 ODs with a single occurrence in the Orange Book, 72 were approved after 31 Dec 2008. e. Of the 72 ODs approved after 31 Dec 2008, 63 had publicly available FDA documents such as the Medical Review. f. The list of 63 ODs, with their year of marketing approval and the FDA document consulted for this study. g. From 2009–2020, per year (i) the number of ODs in this study, (ii) the percentage of ODs, out of 63 (iii) the percentage, in 3-year segments.
(XLS)

S2 File The sources of information (whether MedR or CTG) for the 63 ODs and their 202 trials; the data about each drug and trial extracted from MedR and CTG; and the python script used to web scrape location data from CTG. a. For the 63 ODs, sources of particular drug and trial information. b. For the 63 ODs, the data extracted for each relevant trial from the MedRs, and from CTG where possible*. c. For trials listed in the MedR, the list of matches found in CTG using the trade name, the generic name, or the trial study identifier. d. (a) For trials listed in the MedR, whether the match found in CTG was through the trade name, the generic name, or the trial study identifier; (b) In the trial listing in CTG, the countries listed under Location. e. Python script for web scraping of locations.
(XLS)

S3 File Of the 202 trials, (a) the 9 that ran in India; (b) For the 9 trials in India, a listing of the publications related to each. (c) Of the remaining 193 trials, the 47 (25%) chosen at random to determine whether they might have recruited Indian-origin participants who were based elsewhere. a. For the 9 trials run in India, the following data is provided: NCT ID, All country locations, Drug, Trade Name, Sponsor, Collaborator, Study start date, Study Title, Medical Condition Reported, Participation, Description of participants, and URL. b. For the 9 trials run in India, a list of the publications related to each. c. Of the remaining 193 trials, the 47 (25%) chosen at random to determine whether they might have recruited Indian patients who were based elsewhere. The following data is provided: Trial ID, Number of Asians, Total participants analyzed by race, URL, and Sites in Asia.
(XLS)

S4 File A listing of the World Bank-defined low- and lower-middle income countries, the 11 trials that ran in one or more of these countries, and the 23 publications linked to these 11 trials. a. The World Bank listing of low- and lower-middle income countries. b. The 11 trials that had sites in one or more low- or lower-middle income countries (other than India). c. The 23 publications linked to the 11 trials that had sites in one or more low- or lower-middle income countries (other than India).
(XLS)
